# Tenacissoside H Induces Apoptosis and Inhibits Migration of Colon Cancer Cells by Downregulating Expression of *GOLPH3* Gene

**DOI:** 10.1155/2020/2824984

**Published:** 2020-05-07

**Authors:** Zhong-Shi Hong, Hai-Bin Zhuang, Cheng-Zhi Qiu, Ze-Sheng Shi, Chun-Xiao Wang, Zhi-chuan Chen, Jian-peng Pan

**Affiliations:** Department of General Surgery, The Second Affiliated Hospital of Fujian Medical University, Quanzhou 362000, Fujian Province, China

## Abstract

**Objective:**

Tenacissoside H (TDH) is a Chinese medicine monomer extracted from *Marsdenia tenacissima* extract (MTE), which has been confirmed to have antitumor effects, but its mechanism is still unclear. The aim of this study was to investigate the effect and mechanism of TDH on human colon cancer LoVo cell proliferation and migration and explore the correlation of TDH treatment with the expression of *GOLPH3* and cell signaling pathways in LoVo cells.

**Methods:**

LoVo cells were treated with TDH at 0.1, 1, 10, and 100 *μ*g/mL for 24, 48, and 72 h. The proliferation rate of LoVo cells was evaluated by MTT assay. Recombinant plasmid p-CMV-2-GOLPH3 was constructed, and p-CMV-2-GOLPH3 and p-CMV-2 empty plasmids were transfected into LoVo cells by lipofection. Western blotting was used to detect the transfection efficiency and the expression of p-p70S6K, p70S6K, *β*-catenin, and GOLPH3. The apoptosis rate was analyzed with Annexin V-FITC/PI double-staining method, and cell migration assessed by transwell assay.

**Results:**

TDH inhibited the proliferation of LoVo cells in a concentration-dependent manner. The IC50 of TDH treatment in LoVo cells at 24, 48, and 72 h was 40.24, 13.00, and 5.73 *μ*g/mL, respectively. TDH treatment significantly induced apoptosis and suppressed the viability and migration of human colon cancer LoVo cells. The effect of TDH on induction of apoptosis and inhibition of migration in LoVo cells decreased significantly after activating the PI3K/AKT/mTOR and Wnt/*β*-catenin signaling pathways with agonists. Additionally, the expression of GOLPH3 protein downregulated significantly in LoVo cells under TDH treatment. Overexpression of the GOLPH3 gene increased the expression of key proteins in PI3K/AKT/mTOR and Wnt/*β*-catenin signaling pathways and blocked the antitumor activity of TDH.

**Conclusion:**

Collectively, the present results indicated that TDH can inhibit the proliferation vitality of colon cancer LoVo cells through downregulating GOLPH3 expression and activity of PI3K/AKT/mTOR and Wnt/*β*-catenin signaling pathways.

## 1. Introduction

Colon cancer is one of the most common malignant tumors of the digestive tract. The incidence and mortality rate keep increasing worldwide [1]. Surgical resection is the most important means of treatment at present. However, patients in the advanced stage often received chemotherapy and targeted therapy according to individual conditions. In China, traditional Chinese medicine also plays an important role in the adjuvant treatment of malignant tumors. Studies have shown that many Chinese herbal extracts can inhibit tumor growth and improve quality of life [2]. Through chemical composition analysis and pharmacological studies, it was found that the extract contains different effective chemical ingredients, and the molecular mechanism of its anticancer effect is necessary for further research.

*Marsdenia tenacissima* is the dry vine, root, or leaf of the genus *M. tenacissima* (Roxb.) Wight et Arn, and the isolated *M. tenacissima* extract (MTE) causes obvious inhibitory effect on various malignant cells [3]. Tenacissoside is a kind of major active ingredient in MTE; Tenacissoside H(TDH) is one of its monomers, molecular formula: C_42_H_66_O_14_, belonging to the C21 steroidal glycosides and is considered to be an antitumor active substance isolated from *M. tenacissima* [4]. However, the inhibitory effect and molecular mechanism of TDH on colon cancer is still unfamiliar.

*GOLPH3* has been validated as an oncoprotein and its expression in colon cancer tissues was significantly increased compared to normal tissues [5]; GOLPH3 overexpression can upregulate activation of the PI3K/AKT/mTOR and Wnt/*β*-catenin signaling pathways, promote proliferation, and induce apoptosis in colon cancer cells. Meanwhile, silencing expression of the *GOLPH3* gene can reverse the resistance of HT29 colon cancer cells to 5-fluorouracil and cisplatin [6–8].

It is unclear whether the antitumor effects of TDH are related to downregulation of *GOLPH3* gene expression and cellular signaling pathway. In the present study, we investigated the effect and mechanism of TDH on the proliferation, apoptosis, and migration in human colon cancer LoVo cells.

## 2. Materials and Methods

### 2.1. Drugs and Reagents

The Human colon cancer cell line LoVo (Shanghai Institute of Cell Science, Chinese Academy of Sciences); TDH (20 mg/branch; batch number: 111913-201803; China Food and Drug Control Institute); RPMI1640 medium (Beijing Suolaibao Bioscience and Technology Co., Ltd.); pFLAG-CMV-2 vector (Sigma, St. Louis, MO, USA); cDNA reverse transcription kit (Invitrogen, Carlsbad, CA, USA); Lipofectamine^TM^ 3000 transfection reagent (Invitrogen); Opti-MEM serum-free medium (Shanghai Tuoran Biotechnology Co., Ltd.); rabbit anti-human GOLPH3 polyclonal antibody (ab98023; Abcam, Cambridge, UK); phosphorylated ribosomal protein S6 kinase *β*1 (p-p70S6K) antibody (sc-377529; Santa Cruz Biotechnology, Santa Cruz, USA); p70S6K antibody (sc-8418; Santa Cruz Biotechnology); *β*-catenin antibody (sc-7199; Santa Cruz Biotechnology); horseradish peroxidase-labeled goat anti-rabbit IgG antibody (A0208; Beyotime, Nanking, China); WB and IP protein lysate (Shanghai Lai Maple Biotechnology Co., Ltd.); trypsin powder, 3-(4,5-dimethylthiazol-2-yl)-2,5-diphenyltetrazolium bromide (MTT), dimethyl sulfoxide (DMSO), propidium iodide (PI) staining solution, and fetal bovine serum (FBS) (Sigma); Annexin-V detection kit (Becton Dickinson, Franklin Lakes, NJ, USA); BCA Protein Quantification Kit (Shanghai Weijin Biotechnology Co., Ltd.); Luminol (Shanghai Pu Zhen Biological Technology Co., Ltd.); and tetramethylethylenediamine (TEMED; Beijing BioDee BioTech Corp. Ltd.).

### 2.2. Cell Lines and Culture

The human colon cancer cell line LoVo was purchased from Shanghai Institute of Cell Science, Chinese Academy of Sciences. The LoVo cell line was cultured in RPMI 1640 + 10% FBS and placed in an incubator at 37°C in 5% CO_2_ + 95% air in a saturated humidity environment.

### 2.3. Construction of GOLPH3 Overexpression Plasmid and Cell Transfection

Construction and identification of recombinant plasmid: total RNA in LoVo cells was extracted and reverse transcribed to obtain cDNA, which was subjected to polymerase chain reaction (PCR) amplification (underlined as an enzyme cleavage site) using the following primers: forward, TTTAAGCTTATGACCTCGCTGACCCAGC; reverse, TTTTCTAGATTACTTGGTGAACGCCGCC. The PCR amplification product was a full-length cDNA of 897 bp *GOLPH3* full-length cDNA (NM_022130). The PCR product was digested with *Hin*dIII and *Xba*I and ligated into the pFlag-CMV-2 vector after double digestion. The clones were selected and verified by sequencing to obtain the *GOLPH3* overexpression vector.

Lipofectamine transfection: (1) cells were digested in the exponential phase 1 day before transfection and plated on six-well plates at 10^5^/well; cells were suitable for transfection when they reached 80% confluence; (2) lipofectamine 3000 liposomes were diluted in Opti-MEM and mixed well; (3) empty vector plasmid and recombinant plasmid were diluted with Opti-MEM medium; the premix was prepared and then added with the P3000™ reagent in the kit and mixed well; (4) lipofectamine 3000 liposome was added in a ratio of 1 : 1 with empty vector plasmid and recombinant plasmid premix and was incubated for 5 min at room temperature; and (5) empty plasmid-liposome complexes and recombinant plasmid-liposome complexes were added to the cell culture, and experimental tests were performed.

### 2.4. MTT Assay to Evaluate the Proliferation of LoVo Cells

After blood cell counting, LoVo cells (2 × 10^3^) were inoculated onto 96-well plates at 100 *μ*L/well. After 24 h, different concentrations of TDH were added. At 24, 48, and 72 h after drug treatment, 10 *μ*L 5 mg/mL MTT solution was added to each well and reacted at 37°C for 4 h. Each well added with DMSO and OD490 was determined by using a microplate reader. IC50 was calculated using the Logit method via comparing the OD490 values at different time points.

### 2.5. Cell Grouping

The cells were divided into the following groups: (1) Control, LoVo cells; (2) TDH : TDH-treated LoVo cells; (3) Experimental Group 1, LoVo cells treated with TDH and PI3K/AKT/mTOR signaling pathway agonist insulin-like growth factor (IGF)-1; (4) Experimental Group 2, LoVo cells treated with TDH and Wnt/*β*-catenin signaling pathway agonist Wnt agonist 1; (5) p-CMV-2 : LoVo cells transfected with p-CMV-2 empty plasmid; (6) GOLPH3 : LoVo cells transfected with p-CMV-2-GOLPH3 recombinant plasmid; (7) Experimental Group 3, TDH-treated LoVo cells transfected with empty plasmid; (8) Experimental Group 4: TDH-treated LoVo cells overexpressing GOLPH3 recombinant plasmid. Recombinant plasmid p-CMV-2-GOLPH3 was constructed, and p-CMV-2-GOLPH3 and p-CMV-2 empty plasmids were transfected into human colon cancer LoVo cells by lipofection.

### 2.6. Annexin V-FITC/PI Double-Staining Method to Detect Apoptosis Rate

LoVo cells were digested and seeded in six-well plates (5 × 10^4^ cells). After 24 h, cells were treated with different concentrations of drug according to the experimental group. The treated cells were aspirated in a suitable centrifuge tube, washed once with phosphate-buffered saline (PBS), and the appropriate amount of trypsin cell digest was added. Trypsin-EDTA solution was removed when the adherent cells were obtained. We avoided excessive trypsin digestion. The cell culture solution collected in the abovementioned step was added, mixed, transferred to a centrifuge tube, and centrifuged at 1000 ×*g* for 5 min. The supernatant was discarded and the cells were collected and gently resuspended in PBS and counted. Resuspended cells (50,000–100,000) were centrifuged at 1, 000 ×*g* for 5 min, the supernatant was discarded, and cells were gently resuspended by adding 195 *μ*L Annexin V-fluorescein isothiocyanate (FITC) binding solution. We added 5 *μ*L Annexin V-FITC and mixed gently. We then added 10 *μ*L of propidium iodide (PI) stain and mixed gently. The cells were incubated at room temperature (20°C–25°C) for 10–20 min in the dark and placed in an ice bath. Aluminum foil was used to protect the cells from light. The cells were resuspended two or three times during the incubation to improve the staining effect. Flow cytometry was performed immediately, Annexin V-FITC showed green fluorescence, and PI was red fluorescence.

### 2.7. Transwell Assay for Cell Migration

The LoVo cells were cultured to logarithmic growth and digested, washed, and suspended in serum-free medium, and the concentration was adjusted to 2 × 10^5^ cells; in the lower chamber (i.e., the bottom of the 24-well plate), 600–800 *μ*L culture medium containing 10% serum was added, 100 *μ*L of cell suspension was added to the upper chamber, and different drug treatments were performed according to the experimental group. After continuing culture for 24 h, the chamber was carefully removed with forceps; the upper chamber liquid was blotted and transferred to the well and added with 800 *μ*L methanol, fixed at room temperature for 30 min, rinsed with PBS, and soaked three times. The chamber was then taken out, the upper chamber liquid was removed, and the cells on the surface membrane of the upper chamber bottom with a wet cotton swab were transferred to the lower chamber containing 500 *μ*L of crystal violet and stained at room temperature for 30 min; The cells were observed under an inverted microscope, photographed, and counted.

### 2.8. Western Blotting for Protein Expression

Cells in the logarithmic growth phase were collected and washed once with PBS, lysed in RIPA lysis buffer, and kept on ice for 20 min. The protein concentration was determined by the BCA method, and the total protein of cells extracted by cell lysis was used for SDS-PAGE. After electrophoresis, proteins were transferred to nitrocellulose membranes. PBST containing 5% skimmed milk powder was sealed at room temperature for 60 min. The blots were incubated with rabbit anti-human GOLPH3 polyclonal antibody （1：1000）, p-p70S6K antibody （1：1000）, p70S6K antibody （1：1000）, and *β*-catenin antibody（1：1000） at 4°C overnight and rinsed three times with PBST for 20 min. The corresponding horseradish peroxidase-labeled secondary antibodies were added and incubated for 1 h at room temperature and rinsed three times with PBST for 10 min and detected with ECL chemiluminescence reagent. The image was subjected to gray-scale analysis using Image-J version 1.48 (National Institutes of Health, Bethesda, MD, USA), and the relative intensity of target protein expression is equal to the ratio of the gray value of the target protein band to the gray value of the GADPH band.

### 2.9. Statistical Analysis

Statistical analysis was performed using SPSS version 20.0. All samples were tested to ascertain if they followed the homogeneity of variance. The paired *t*-test was used for comparison between two groups and one-way analysis of variance was used for comparison between ≥3 groups. The least significant difference method was used for multiple comparisons between groups, and *P* <0.05 indicated that the difference was statistically significant.

## 3. Results

### 3.1. TDH Inhibits the Proliferation of LoVo Cells

**Figure 1:**
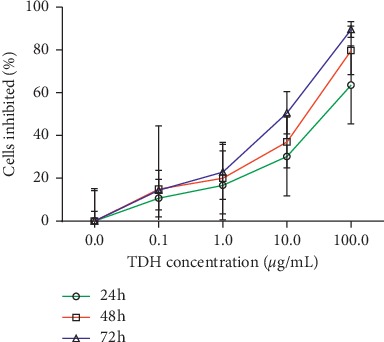
Inhibition of LoVo cells proliferation under different concentrations of TDH treatment. The IC50 of TDH in LoVo cells at 24, 48, and 72 h was 40.24, 13.00, and 5.73 *μ*g/ml.

MTT assay was used to detect the LoVo cell proliferation under TDH treatment at concentrations of 0.1, 1, 10, and 100 *μ*g/ml for 24, 48, and 72 h, and the respective growth inhibition curves were drawn (Figure 1). The results showed TDH inhibited significantly LoVo cell proliferation in a concentration-dependent manner (Table 1). However, under the same TDH concentration, there was no significant difference in the inhibitory effect of TDH on LoVo cell proliferation as the treatment time was prolonged (*P* > 0.05). It suggested that there was no obvious time-dependent effect of TDH. The IC50 of TDH treatment in LoVo cells at 24, 48, and 72 h was 40.24, 13.00, and 5.73 *μ*g/ml, respectively. In the subsequent experiments, the concentration of TDH was selected to be 25 *μ*g/ml.

### 3.2. TDH Induces Apoptosis of LoVo Cells

**Figure 2:**
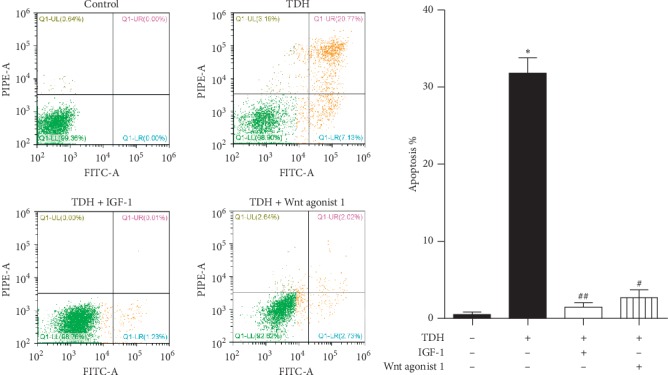
Apoptosis rate of LoVo cells under TDH treatment. Comparison of the TDH Group with the Control Group (^*∗∗*^*P* < 0.01). Comparisons of Experimental Group 1 and Experimental Group 2 with the TDH Group (^#^*P* < 0.01, ^##^*P* < 0.01).

Apoptosis was detected by Annexin V-FITC/PI flow cytometry. The apoptosis rates of the Control Group, the TDH Group, Experimental Group 1, and Experimental Group 2 were 0.51 ± 0.54%, 31.77 ± 3.47%, 1.47 ± 0.97%, and 2.68 ± 1.79%, respectively. The apoptosis rate of the TDH Group was significantly higher than the Control Group (*P* < 0.01). The apoptosis rate of Experimental Group 1 and Experimental Group 2 was significantly lower than the TDH Group (*P* < 0.05) (Figure 2). This result indicated that the PI3K/AKT/mTOR or Wnt/*β*-catenin signaling pathway agonist could attenuate the apoptosis induced by TDH in LoVo cells.

### 3.3. TDH Inhibits LoVo Cell Migration

**Figure 3:**
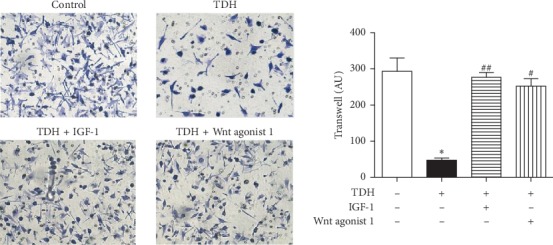
LoVo cell migration under TDH treatment. Comparison of the TDH Group with the Control Group (^*∗*^*P* < 0.05). Comparisons of Experimental Group 1 and Experimental Group 2 with the TDH Group (^#^*P* < 0.05, ^##^*P* < 0.01).

The numbers of cell migration in the Control Group, the TDH Group, Experimental Group 1, and Experimental Group 2 were 293 ± 64, 47 ± 12, 277 ± 23, and 253 ± 35, respectively (Figure 3). The migrated cells in the TDH Group were significantly decreased than the Control Group (*P* < 0.05). Meanwhile, compared with the TDH Group, the migrated cells in Experimental Group 1 and Experimental Group 2 increased significantly (*P* < 0.05). The experiments showed that signal pathway agonists could reduce the inhibitory effect of TDH on migration in LoVo cells.

### 3.4. TDH Affects LoVo Cell Signaling Pathway-Related Protein Expression

**Figure 4:**
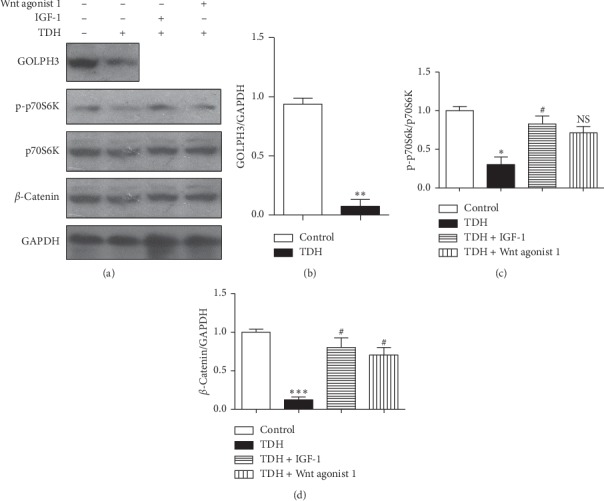
(a) Expression of related proteins in LoVo cells under TDH treatment. (b) The TDH Group compared with the Control Group (^*∗*^*P* < 0.05, ^*∗∗*^*P* < 0.01, ^*∗∗∗*^*P* < 0.001); (c) Experimental Group 1 and (d) Experimental Group 2 compared with the TDH Group (NS *P* < 0.05, ^#^*P* < 0.05).

Compared with the Control Group, expression of p-p70S6K and *β*-catenin in the TDH Group was significantly decreased (*P* < 0.05), and the corresponding signaling pathway was inhibited (Table 2, Figure 4). After adding the signaling pathway agonist (IGF-1 or Wnt agonist 1), expression of p-p70S6K in Experimental Group 1 and *β*-catenin expression in Experimental Group 2 were significantly higher than in the TDH Group (*P* < 0.05). Moreover, under TDH treatment, the GOLPH3 expression in LoVo cells was also altered, and the expression of GOLPH3 protein in the TDH Group was significantly lower than in the Control Group (*P* < 0.01). These results demonstrated that TDH could downregulate both GOLPH3 expression and activation of the PI3K/AKT/mTOR and Wnt/*β*-catenin signaling pathway in LoVo cells.

### 3.5. Effect of TDH on the Expression of Signaling Pathway-Related Proteins in LoVo Cells Overexpressed GOLPH3

**Figure 5:**
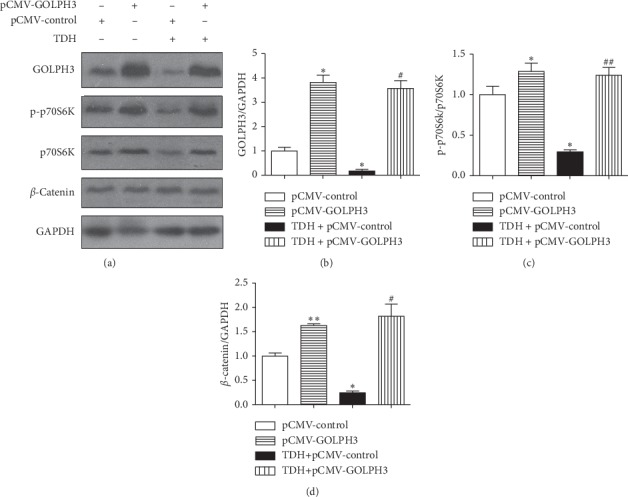
(a) Expression of related proteins in LoVo cells overexpressed GOLPH3 under TDH treatment. (b) The GOLPH3 Group and (c) Experimental Group 3 compared with the p-CMV-2 Group (^*∗*^*P* < 0.05, ^*∗∗*^*P* < 0.01); (d) Experimental Group 4 compared with Experimental Group 3 (^#^*P* < 0.05, ^##^*P* < 0.01).

Compared with the p-CMV-2 Group, expression of GOLPH3 protein in the GOLPH3 Group increased significantly (*P* < 0.05); it indicated that the transfection efficiency was good (Table 3, Figure 5). Moreover, expression of p-p70S6K, *β*-catenin, and GOLPH3 proteins in Experimental Group 4 was increased significantly than Experimental Group 3 (*P* < 0.05). It implied that GOLPH3 overexpression can hinder the inhibitory effect of TDH on PI3K/AKT/mTOR and Wnt/*β*-catenin signaling pathways in LoVo cells.

### 3.6. Effect of TDH on Apoptosis in LoVo Cells Overexpressed GOLPH3

**Figure 6:**
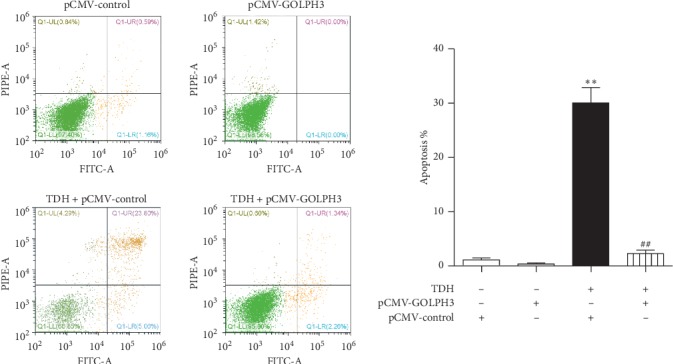
Apoptosis rate of LoVo cells overexpressed GOLPH3 under TDH treatment. Experimental Group 3 compared with the p-CMV-2 Group (^*∗∗*^*P* < 0.01); Experimental Group 4 compared with Experimental Group 3 (^##^*P* < 0.01).

Apoptosis was detected by Annexin V–FITC/PI flow cytometry. Apoptosis rates in the p-CMV-2 Group, the GOLPH3 Group, Experimental Group 3, and Experimental Group 4 were 1.11 ± 0.65%, 0.34 ± 0.35%, 30.06 ± 4.86%, and 2.27 ± 1.19% (Figure 6). The apoptosis rate of Experimental Group 3 was significantly higher than the p-CMV-2 Group, while the apoptosis rate of Experimental Group 4 was significantly lower than Experimental Group 3 (*P* < 0.01). This result testified that GOLPH3 overexpression in LoVo cells could arrest the TDH-induced apoptosis.

### 3.7. Effect of TDH on Migration in LoVo Cells Overexpressed GOLPH3

**Figure 7:**
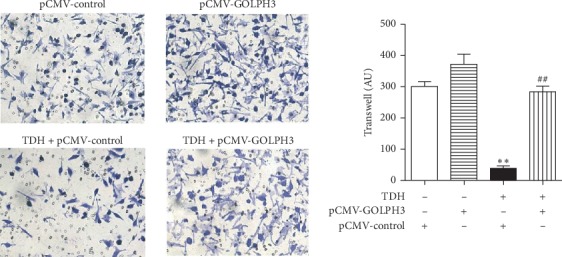
Effect of TDH on migration in LoVo cells overexpressed GOLPH3 under TDH treatment. Experimental Group 3 compared with the p-CMV-2 Group (^*∗∗*^*P* < 0.01); Experimental Group 4 compared with Experimental Group 3 (^##^*P* < 0.01).

The numbers of cell migration in the p-CMV-2 Group, the GOLPH3 Group, Experimental Group 3, and Experimental Group 4 were 301 ± 26, 372 ± 55, 39 ± 14, and 284 ± 31, respectively(Figure 7). The number of migrated cells in Experiment Group 3 was significantly decreased than the p-CMV-2 Group (*P* < 0.01). Correspondingly, the number of migrated cells in Experimental Group 4 was also significantly higher than that in Experimental Group 3 (*P* < 0.01). The experiments manifested that the inhibitory effect of TDH on migration in LoVo cells overexpressed GOLPH3 significantly weakened.

## 4. Discussion

In China, the incidence and mortality of colorectal cancer continue to rise [9]. Although chemotherapy and targeted drugs can improve the survival rate, they are expensive and have obvious adverse effects. To improve the life quality of patients with advanced colon cancer by comprehensive treatment, traditional Chinese medicine is used to assist patients with colon cancer. It can effectively reduce the adverse effects of chemotherapy, improve immunity, and play an important role in the treatment of colon cancer [10]. *M. tenacissima* is widely used as a traditional Chinese medicine in clinical practice; its preparation Xiaoaiping is used for adjuvant treatment in various malignant tumors; it was interesting to note that the therapeutic effect is remarkable and the adverse reactions are few [11–13]. The composition of *M. tenacissima* is complex, and its antitumor mechanism is still unclear and needs further study.

Related studies have shown that TDH can inhibit the proliferation of esophageal cancer cells by regulating the cell cycle and reducing proliferating cell nuclear antigen expression [14]. TDH can also inhibit the growth of mouse lung cancer cells, reduce the number of lung metastases, and enhance the immune function of tumor-bearing mice by regulating Th1/Th2-related cytokines [15]. We found that TDH had a significant concentration-dependent inhibitory effect on the proliferation of human colon cancer LoVo cells *in vitro*; moreover, it can also induce apoptosis and inhibit the migration of LoVo cells; these results indicated that TDH could inhibit colon cancer cell viability and metastasis. The abnormal activation of the PI3K/AKT/mTOR signaling pathway has been observed in a variety of human tumors, including esophageal, ovarian, breast, and colorectal cancer and promoted the development of tumor by promoting cell proliferation, inhibiting apoptosis, or activating angiogenesis [16–19]. The aberrant activation of the Wnt/*β*-catenin signaling pathway has been also demonstrated in colorectal cancer and regulates gene expression and cell invasion, migration, proliferation, and differentiation in the initiation and progression of CRC [20]. In the present study, under TDH treatment, the expression of p-p70S6K and *β*-catenin was significantly reduced in LoVo cells; however, the expression of p-p70S6K and *β*-catenin was upregulated, apoptosis decreased, and cell migration increased after using mTOR and Wnt signaling pathway agonists; the result suggested that TDH may exert antitumor activity through inhibiting the PI3K/AKT/mTOR and Wnt/*β*-catenin signaling pathways in the colon cancer.

In addition, we also found that the decrease of GOLPH3 expression was detected in LoVo cells under TDH treatment. GOLPH3 is localized in the Golgi apparatus, widely involved in many important physiological processes, and plays an important role in glycosyltransferases location, glycosylation, and secretory trafficking which were related to cancer development. *GOLPH3* is a oncogene that is frequently overexpressed in various malignant tumor tissues, including glioma and liver, lung, ovarian, and esophageal cancer, and is positively correlated with poor prognosis [21–25]. It is also reported that GOLPH3 overexpression can activate various signaling pathways, promote cell proliferation, inhibit apoptosis, and induce the sensitivity of colon cancer cells to cisplatin and 5-FU [7, 8]. In the present study, under the same condition of TDH treatment, compared with nontransfected LoVo cells, p-p70S6K and *β*-catenin expression and cell migration increased in LoVo cells overexpressed *GOLPH3*; these results suggested that the antitumor effect of TDH on LoVo cells was related to inhibition of the PI3K/AKT/mTOR and Wnt/*β*-catenin signaling pathways through downregulating *GOLPH3* gene expression. Therefore, we can reasonably speculate that TDH can block the resistance of colon cancer cells to cisplatin and 5-FU and improve chemotherapeutic efficacy in patients with advanced colon cancer.

## 5. Conclusion

TDH can inhibit the proliferation and migration and induce apoptosis in human colon cancer LoVo cells. The mechanism is that TDH can inhibit the PI3K/AKT/mTOR and Wnt/*β*-catenin signaling pathways by downregulating *GOLPH3* gene expression.

## Figures and Tables

**Table 1:** Proliferation inhibition rates of LoVo cells under TDH treatment.

TDH concentration	Proliferation inhibition rate (%)		
(*μ*g/mL)	24 h	48 h	72 h
0	0.00 ± 15.06	0.00 ± 4.53	0.00 ± 14.22
0.1	10.72 ± 8.78 NS	14.98 ± 29.49 NS	14.42 ± 9.19 NS
1	16.74 ± 16.20 NS	20.00 ± 16.76 NS	22.96 ± 12.80^*∗∗*^
10	30.17 ± 18.43^*∗*^	37.00 ± 12.22^*∗∗*^	50.62 ± 9.88^*∗∗∗*^
100	63.61 ± 18.27^*∗∗∗*^	79.81 ± 11.34^*∗∗∗*^	89.63 ± 3.64^*∗∗∗*^

Results are shown as mean ± SD. Comparisons of different concentrations in the same time (NS *P* > 0.05, ^*∗*^*P* < 0.05, ^*∗∗*^*P* < 0.01, ^*∗∗∗*^*P* < 0.001). 24 h, *F* = 9.725; 48 h, *F* = 12.959; 72 h, *F* = 44.726.

**Table 2:** Expression levels of proteins under TDH treatment.

Group	p-p70S6K/p70S6K	*β*-Catenin
Control	1.00 ± 0.10	1.00 ± 0.06
TDH	0.30 ± 0.17^*∗*^	0.12 ± 0.06^*∗∗∗*^
TDH + IGF-1	0.83 ± 0.18^#^	0.80 ± 0.22^#^
TDH + Wnt agonist 1	0.71 ± 0.15 NS	0.70 ± 0.17^#^

Results are shown as mean ± SD. The TDH Group compared with the Control Group (^*∗*^*P* < 0.05, ^*∗∗∗*^*P* < 0.001); Experimental Group 1 and Experimental Group 2 compared with the TDH Group (NS *P* > 0.05, ^#^*P* < 0.05).

**Table 3:** Expression levels of proteins in LoVo cells overexpressed GOLPH3 under TDH treatment.

Group	p-p70S6K/p70S6K	*β*-Catenin	GOLPH3
pCMV-control	1.00 ± 0.18	1.00 ± 0.11	1.00 ± 0.27
pCMV-GOLPH3	1.29 ± 0.18^*∗*^	1.63 ± 0.06^*∗∗*^	3.82 ± 0.52^*∗*^
TDH + pCMV-control	0.29 ± 0.05^*∗*^	0.24 ± 0.07^*∗*^	0.17 ± 0.12^*∗*^
TDH + pCMV-GOLPH3	1.24 ± 0.17^##^	1.82 ± 0.44^#^	3.57 ± 0.55^#^

Results are shown as mean ± SD. The GOLPH3 Group and Experimental Group 3 compared with the p-CMV-2 Group (^*∗*^*P* < 0.05, ^*∗∗*^*P* < 0.01); Experimental Group 4 compared with Experimental Group 3 (^#^*P* < 0.05, ^##^*P* < 0.01).

## Data Availability

The data used to support the findings of this study are available from the corresponding author upon request.
